# Exclusive breastfeeding in first-time mothers in rural Kenya: a longitudinal observational study of feeding patterns in the first six months of life

**DOI:** 10.1186/s13006-020-00260-5

**Published:** 2020-03-05

**Authors:** Alison Talbert, Caroline Jones, Christine Mataza, James Alexander Berkley, Martha Mwangome

**Affiliations:** 1 Kenya Medical Research Institute/Wellcome Trust Research Programme, Kilifi, Kenya; 2 Centre for Tropical Medicine and Global Health, Oxford University, Oxford, UK; 3 Kilifi County Department of Health, Kilifi, Kenya; 4 The Childhood Acute Illness & Nutrition Network (CHAIN), Nairobi, Kenya

**Keywords:** Breastfeeding, Exclusive, Barriers, Kenya, Mothers, Observation, Infant, Feeding, First-time

## Abstract

**Background:**

Exclusive breastfeeding up to 6 months of age is recommended by the World Health Organization as the optimal mode of infant feeding, providing adequate nutrition for the baby and protection against infectious diseases. Breastfeeding can be adversely affected by individual, cultural and socio-economic factors. The study aimed to explore barriers of exclusive breastfeeding in the first 6 months of life among first-time mothers in rural Kenya.

**Methods:**

An observational longitudinal design aimed to provide rich data on breastfeeding behaviour. Twenty pregnant first-time mothers were recruited through antenatal clinics and snowballing. Mothers were visited nine times at home from late pregnancy, at 1 week and 2 weeks post-delivery, then monthly until the baby was aged 6 months. Visits were conducted between November 2016 and April 2018. At the first visit, participants were asked about breastfeeding intentions and infant feeding education received. At each postnatal visit, direct observation of breastfeeding, a recorded semi-structured interview on feeding, mother’s and baby’s health was performed. Interviews were transcribed, checked, content was grouped into categories and analyzed using a qualitative descriptive approach.

**Results:**

Most participants were adolescent (75%) and unmarried (65%). All 20 mothers intended to and did breastfeed, however additional fluids and semi-solids were commonly given. Only two mothers exclusively breastfed from birth up to 6 months of age. Prelacteal feeds, home remedies and traditional medicine were given by over a third of mothers in the first week of life. Concern over babies’ bowel habits and persistent crying perceived as abdominal colic led to several mothers receiving advice to give gripe water and traditional remedies. Early introduction of maize porridge from 3 months of age because of perceived hunger of the child was recommended by other family members. Breastfeeding observation showed persistent problems with positioning and attachment of infants.

**Conclusions:**

Exclusive breastfeeding from birth to 6 months was uncommon. Prioritization of capacity to detect mothers with breastfeeding problems and provide breastfeeding education and support is necessary, particularly during the antenatal and early postnatal period. It is important to engage with other women resident in the household who may offer conflicting feeding advice.

## Background

Exclusive breastfeeding up to 6 months of age is recommended by the World Health Organization as the optimal mode of infant feeding, providing adequate nutrition for the baby together with protection against respiratory infections and diarrhoeal diseases [1–5]. Despite its benefits, the practice of breastfeeding can be adversely affected by many factors working at different levels: political, socio-economic, cultural and individual including both mother and baby [6].

Estimates of the prevalence of exclusive breastfeeding (EBF) in infants under 6 months old made by the national demographic and health surveys in Kenya have increased between 2008 and 2014 from 32 to 61% [7, 8]. Researchers have however cautioned that cross-sectional surveys based on 24-h recall over-estimate the percentage of infants on EBF from birth to 5 months [9, 10]. This is because infants may receive other fluids and foods intermittently before returning to EBF. These figures also represent an average prevalence of breastfeeding across this age range. The proportion of babies still EBF in Kenya in the 4 to 5 months age group is lower at 42% [7].

The multi-country ‘Etiology, Risk Factors and Interactions of Enteric Infections and Malnutrition and the Consequences for Child Health and Development’ (MAL-ED) study on malnutrition and enteric infections in Africa, South Asia and South America reported that the prevalence of EBF in the first month of life was less than 60%, and partial breastfeeding (BF) over 20% in 6 out of 8 sites [11]. Risk factors for early interruption were identified as prelacteal feeding, discarding of colostrum and being a first-time mother. A recent mixed-methods study of 50 first-time mothers and their advisers in a rural coastal community in Kenya described multiple problems in practicing breastfeeding in the first month of life [12].

Becoming a mother is seen as a mark of entry into adulthood and a fulfilment of social expectations in different African contexts [13, 14]. However when pregnancy occurs in adolescence there may be added stress in taking on adult responsibilities before the girl is adequately prepared [15]. Lack of financial independence, disruption of education and uncertainty over support from the baby’s father may lead to concerns about material provision and social support for mother and child during this time of transition of roles [16]. There may also be a failure of health services to cater for the emotional and mental health needs of pregnant adolescents during antenatal and maternity care [17].

Adolescent pregnancy rates are high in sub-Saharan Africa with multiple social determinants including poverty, unequal gender power relations, transactional sex, absence of affordable education and lack of parental guidance [18]. Pregnancy in adolescence is associated with an increased rate of preterm birth and malnutrition in the offspring [19]. A study in Western Kenya found that young mothers under 20 years old had a shorter duration of exclusive breastfeeding than older cohorts [20]. In coastal Kenya 21% of women aged 15 to 19 years old have begun childbearing [7] however there is little published information from this area on the first-time mothers’ practice of exclusive breastfeeding.

The current study was designed to explore first-time mothers’ trajectories of feeding practices from birth until the baby was 6 months old. In the study the main research question was “What are the modifiable barriers to exclusive breastfeeding in the first six months of life among first-time mothers living in a rural area of coastal Kenya?” Specific objectives of the study were to: understand perceptions of breastfeeding and intentions to practice from late pregnancy until the infant is 6 months of age; to assess breastfeeding technique and exclusivity of breastfeeding; to identify the key actors influencing breastfeeding practice and explore the challenges and enablers of exclusive breastfeeding. The aim of this formative research is to assist in the design of contextualized interventions for enabling exclusive breastfeeding from birth up to 6 months of age. This paper describes the perceptions, intentions and practices of breastfeeding and gives a detailed description of the factors affecting exclusivity of breastfeeding at the different timepoints of follow up.

## Methods

### Study design and location

An observational longitudinal design was chosen to study breastfeeding behaviour in a small group of women followed up intensively from late pregnancy until 6 months after delivery. The sample size of 20 was chosen pragmatically in line with guidance for a focused ethnographic study design involving purposive selection of a small sample to collect information-rich data from a specific population [21]. The study population was women living in two administrative locations, with a total population of approximately 25,000, in a rural area 20 km from the nearest town. The population is served by two government dispensaries where normal deliveries are carried out except at weekends. Previous research on infant feeding had been carried out in the area with good cooperation from local administrative chiefs, health workers and community members. The Kilifi county health management team, health facility staff and local community leaders were briefed on the aims and methods of this study and gave their authorization prior to commencement.

The study site was in an area with a semi-arid climate with high levels of poverty and food insecurity. Subsistence farming was the main occupation (39%) with 21% in paid employment [22]. Education levels were low, with 52% of adult Kilifi county residents having completed primary education [22]. The teenage pregnancy rate in Kilifi county was amongst the highest in the country with 21.8% of 15–19 year old girls pregnant or having given birth [7].

### Ethical approval

The study received ethical approval from the Kenya Medical Research Institute Scientific and Ethics Review Unit (KEMRI/SERU/CGMR-C/0033/3228).

### Sampling and consent procedures

Sampling was purposive to recruit a cohort of 20 first-time mothers resident in the study area. Nurses in the two government health facilities were asked to assist in identifying pregnant women who might be eligible for the study. Enrolment was a two-stage process. In the first stage, women attending antenatal clinic in the third trimester of pregnancy were given information about the aims of the study and asked if they would be interested to participate. If they agreed, then the research assistant asked if she could visit them in their homes to complete the consenting process. This gave the women the opportunity to return home and consult with other family members. The second stage involved the research assistant visiting the homestead to request written informed consent from the woman to participate in the study and request permission from the household head, to allow the study activities to take place there.

### Data collection

There were nine home visits for each mother, the first being towards the end of pregnancy. The participants were then asked to inform the researchers soon after giving birth so that the postpartum visit could be conducted when the baby was 1 week old. Subsequent visits were at 2 weeks post-delivery, then at the end of each month of life until the baby completed 6 months. We scheduled visits for the convenience of families and so that the participants would be at home.

Data collection was carried out during home visits. The researchers carrying out the home visits were both female, a British doctor who was fluent in Kiswahili and a trained research assistant from the study community with secondary education who was fluent in the local language Kikauma as well as Kiswahili and English. Participants all spoke Kiswahili. The researchers introduced themselves to family members on arrival at the homestead then arranged to sit in a private place to interview the mother away from others and observe breastfeeding technique. The interviews were recorded on a digital recorder for later transcription. Researchers wrote field notes after each visit.

Two observers watched at least one breastfeeding episode per visit to check for signs of possible difficulty, according to the WHO/UNICEF breastfeeding observation aid (BOA) [23] (see Additional file 1). The BOA comprises a checklist of 22 dichotomous variables organized into six categories: mother, baby, breast, baby’s position, baby’s attachment, suckling. A score was obtained by allocating one point for each problem observed out of a total of 22. Total scores per mother per visit were obtained as well as scores for each category.

Semi-structured interviews were carried out using interview guides covering family relationships, food security, infant feeding intentions and practices, and mother and baby health (see Additional file 2). Questions on breastfeeding included if the baby had been breastfed and if any other fluids or foods had been given since the previous study visit and reasons why. Mothers were asked about the frequency of breastfeeds, wet nappies and stools in the last 24 h. Mothers and babies found to have health or breastfeeding problems requiring treatment were advised to seek help from local health providers and in some cases assisted with transport and referral.

Recordings of interviews were transcribed by the principal investigator and two data entry clerks. The transcripts were checked for content against the audio recordings by the research assistant. The transcripts were copied onto NVivo 10 (QSR International) software for coding.

### Classification of feeding practices

Exclusive breastfeeding was defined as giving breast milk only except for oral vaccines, vitamins, minerals and prescribed oral medicines [24]. Predominant breastfeeding allowed addition of water and water-based fluids, whereas partial breastfeeding included non-human milks such as cow’s milk and formula milk. Complementary feeding was defined as any semi-solid or solid food given in conjunction with breastfeeding. The mothers’ responses on feeding practice since the previous interview were used to classify the mode of feeding. The timing and reasons for departing from exclusive breastfeeding were sought.

### Qualitative descriptive analysis of interviews

A coding framework was designed by the principal investigator deductively from the topics from the interview guides. The transcripts were read and analyzed for content by the principal investigator then codes were assigned to sections of the data according to the coding framework. Coded data was organized into three main groups 1) mother and baby: feeding, health; 2) family and social support; 3) education and work outside the home to help to differentiate the levels of individual, interpersonal and community/environment influence. These groups were used to investigate individual and common explanations for suboptimal feeding practices taking into consideration the age of the child. Discussions with a second investigator helped to build consensus around the interpretation of the data.

## Results

Recruitment of 20 study participants took place between November 2016 and September 2017, and the last follow up visit was in April 2018. One participant withdrew after the 3 months visit on the advice of her sister-in-law so that 19 women completed follow up to the 6 months visit. The researchers recorded 172 interviews with mothers.

The median age of the participants was 17 years (range 15–26 years) and the majority were single at time of enrolment (Table 1). One mother was the second wife in a polygamous household. Only half of mothers had completed 8 years of primary school. Reasons cited for dropping out were pregnancy and inability to pay school costs. Single participants mostly lived with their mothers but in two households their mothers were working away from home and in another two families, participants lived with their grandmothers. During the follow up period one participant moved to live with the baby’s father, and one married woman separated from her husband and moved back to her parents’ house.

**Table 1:** Participants’ demographic and household characteristics

	No. of participants (%)
**Age of mother**	
15–29	15 (75)
20–24	4 (20)
25–29	1 (5)
**Highest level of education achieved**	
College diploma	1 (5)
College entry	1 (5)
Secondary school not completed	3 (15)
Primary school certificate	4 (20)
Primary school not completed	11 (55)
**Marital status at enrolment**	
Single	13 (65)
Married	6 (30)
Separated from husband	1 (5)
**Co-residence**	
Living with mother	10 (50)
Living with husband and mother-in-law	4 (20)
Living with grandmother	2 (10)
Living with siblings	1 (5)
Living with maternal aunt	1 (5)
Living with husband, mother-in-law is absent	1 (5)
Living with mother-in-law, husband is absent	1 (5)
**Financial support from baby’s father**	
Yes	15 (75)
No	5 (25)

### Birth place

Seven births took place at home, three in the local government dispensary, four in the local private clinic, one in a government health centre in the adjoining location, four in the county hospital and one in a private hospital. Two mothers required emergency Caesarian section for prolonged labour and one of these mothers suffered the stillbirth of her second twin.

### Infant feeding intentions

During the first visit in late pregnancy mothers were interviewed about their plans for feeding their infant. All intended to breastfeed, though four women couldn’t say for how long. One woman said she would breastfeed for 6 months and another for 9 months. Three said until the baby was walking and six women until the child was aged 2 years or above. Two mentioned that they would give cow’s milk in the first 6 months, one because she intended to stop breastfeeding and return to boarding school at 2 months and the other to supplement breast milk. One mother planned to go back to school 1 month after the birth and said she hadn’t thought how the baby would be fed then. Most said they would introduce soft food such as porridge and bananas at 6 to 8 months although two did not know when.

### Early breastfeeding and skin-to-skin contact

Mothers delivering at the county hospital reported that they were given the baby to hold immediately after birth for a few minutes but actually started breastfeeding only after being transferred to the postnatal ward 1 h post-delivery. Those who gave birth in local dispensaries, whether government or private, said they were discharged within 1 to 2 h after the birth and did not breastfeed until they reached home. Some mothers who delivered at home reported breastfeeding within 1 h of birth but there were also mothers who did not breastfeed until the following day. In home births the mother was bathed and allowed to rest before being presented with the baby to breastfeed.

### Frequency of breastfeeds per 24 h

It was difficult for some mothers to specify exact numbers of breastfeeds in the preceding 24 h despite researchers requesting this information at each visit. We tried to assess the frequency of BF because infrequent feeds may have been an indicator that the baby is not receiving sufficient opportunities to breastfeed; the mother may not be aware of the baby’s demands, she is too busy or she may not be around. We asked mothers to describe cues that the baby was hungry but most only mentioned crying. We also realized that some mothers do not know how often a baby should be fed and would let them sleep overnight in the first few days of life. At the end of the first week the number of breastfeeds was less than eight per day in six out of 20 mothers.

### Signs of difficulty observed during breastfeeding

The median total score per mother from breastfeeding observations fell from six (IQR 4.3 to 7.5) at the week one visit to three (IQR 2 to 3) at 6 months. Breast pain was reported by eight mothers at the week one visit. The most common problems were in positioning baby at the breast: not holding the baby close (19 mothers at week one to 14 at month six) and not approaching nose to nipple (13 mothers at week one and 19 at month six) and attachment: the baby’s mouth was not open wide (11 at week one and none at month six), and chin not touching the breast (nine mothers at week one and month six visits).

### Mode of breastfeeding

**Fig. 1:**
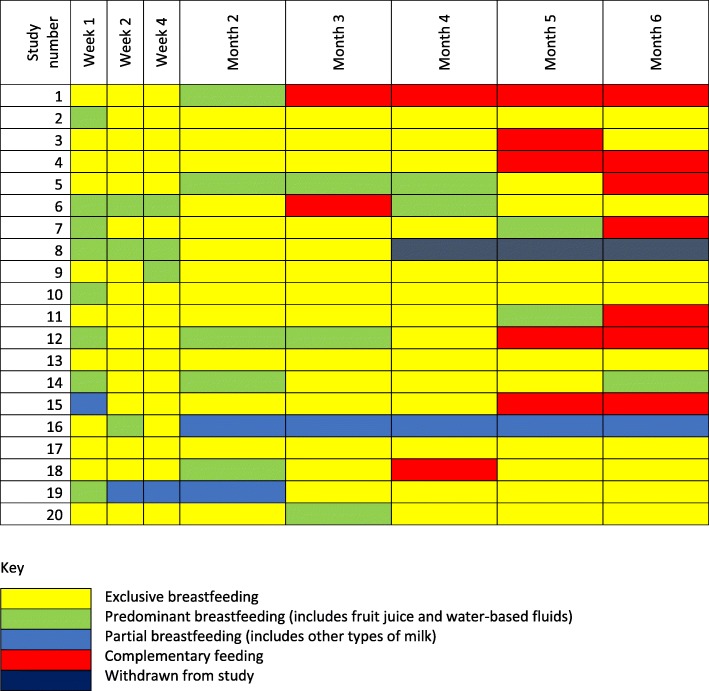
Mode of feeding reported by mothers at study visits

Figure 1 shows the feeding patterns of individual mothers revealing the time intervals when additional fluids or solids are most likely to be introduced.

#### Exclusive breastfeeding

The prevalence of EBF, defined as giving only breast milk and no extra fluids or solids since the previous study visit, was lowest in the first month of life 45% (9/20 mothers) and highest in the third month 70% (14/20) (Fig. 1). Only two (10.5%) mothers practiced this from birth to 6 months. There were no obvious features to distinguish those mothers who exclusively breastfed from those who did not. These two mothers, participants 13 and 17, were both adolescent and had dropped out of primary school. One was married and living with her husband in his parents’ homestead, the other was single and living with her grandmother. Both gave birth in government primary health care facilities.

#### Predominant breastfeeding

Predominant breastfeeding occurred most commonly in the first month of life 45% (9/20 mothers) when prelacteal feeds and traditional medicines were given. Three mothers gave prelacteal feeds. One mother’s husband brought the baby some coconut water on the first day on the instruction of his mother. The reason given was that the baby had not started sucking. Another mother gave sugar solution on her own initiative on the first day because she was concerned that the breastmilk volume was insufficient. A third mother gave sugar solution because the baby was irritable and refused to suck.

In the first week traditional medicine was given by mouth to two babies to treat episodes of crying perceived to be due to abdominal pain, one baby for nasal secretions, and one baby because of startling at night and jaundice. Another baby was given a home-made sugar and salt solution on days two and three of life to stimulate bowel movements. These treatments were suggested by the participants’ mother in laws. Another participant gave traditional medicine in the second week because her baby was crying at night. This was thought to be best treated by treatment from a traditional healer rather than prescribed medicine.

Gripe water was given particularly in the first month but also in the second month as a treatment for perceived abdominal pain as shown by crying. Gripe water, an Ayuverdic herbal mixture containing dill seed oil, was bought over the counter from local shops. The mother’s mother or mother-in-law advised it but two mothers reported they gave it on the recommendation of a health worker.

Non-milk fluids such as water and orange juice were given from the second month of life. Reasons for giving additional water were the baby was perceived to be thirsty, to cool the baby because “the sun is fierce” and because the mother’s milk was hot. For one baby the juice from half an orange was given every day from month two to “loosen his bowels”. As babies grew, mothers reported that they showed interest when adults were drinking so they gave some water or tea for them to try. One mother started giving her baby water to drink because he was lapping from the basin whilst being bathed.

#### Partial breastfeeding

Partial breastfeeding with breastmilk substitutes was related to feeding and medical problems in two babies which were resolved in the first 2 months. A third mother returned to school and the caretaker supplemented with cow’s milk from when the baby was 2 months old.

The baby of mother 19 experienced difficulty in feeding throughout the first month after a home birth with meconium stained liquor. The infant was unsettled from birth and unable to suck so the mother sought advice from family and neighbours, feeding the baby on sugar solution on the second and third days of life and giving orange juice to open the bowels. In addition, the baby received several prescribed medicines including antibiotics and an antispasmodic agent from a private clinic in the first week of life. Despite an attempt by the researchers to teach the mother how to give expressed breastmilk until the baby could suck, the mother tried to breastfeed, and also bought full cream milk powder to supplement. The baby was admitted to hospital with severe acute malnutrition at 30 days of age where they were treated with therapeutic milk feeds (dilute F100) and antibiotics and given relactation support. The baby subsequently made a good recovery on exclusive breastfeeding achieving normal anthropometric measurements by 5 months of age.

Baby 15, a first twin with low birthweight, whose mother had an emergency Caesarian section for a retained second twin, received formula milk in hospital after developing a fever and low blood sugar on the second day of life. The mother’s mental state following the stillbirth of the second twin impacted on her ability to breastfeed whilst in hospital and the nurses supplemented feeds with formula milk.

One teenage mother returned to primary school when the baby was 2 months old. She lived half a kilometre from the school. She returned home at mid-morning break and lunchtime to breastfeed but her mother who was babysitting also supplemented with cow’s milk.

Another teenage mother returned to secondary school when the baby was 4 months old, having originally intended to return 1 month after the birth. She walked an hour each way to school, was away from home for over 12 h a day and her mother looked after the baby in her absence. She reported that she left expressed breastmilk for the baby but said she could not know what her mother gave if she was not there.

#### Early introduction of complementary feeding

Complementary feeds were introduced alongside breastfeeding from month three onwards by nine mothers but two babies refused them so they were discontinued. From 3 months of age, these mothers gave maize porridge, sometimes with sugar and margarine. It was generally given in the morning only. The most common explanation for starting maize porridge was the baby crying after breastfeeding which was interpreted as the baby getting insufficient milk so that the baby’s grandmother suggested giving porridge.

One mother was evicted by a co-wife when the baby was 3 months old and moved back to the parental home. She gave porridge on her mother’s advice soon afterwards because she said stress had adversely affected her milk production. When her milk production improved she stopped regular porridge, but gave it intermittently when her milk supply seemed low. Another mother reported giving porridge during a febrile illness when her milk supply was temporarily reduced.

One mother, a primary school teacher, reported that the baby’s crying was preventing her from doing the housework so she gave porridge when the baby was 5 months old. She said she had been advised by people “from all around” to start porridge and when she told them that clinic advice was to wait until 6 months she replied they had told her “then he will continue to disturb you.”

## Discussion

Our longitudinal study of infant feeding experiences by twenty first-time rural mothers showed that all intended to breastfeed and were still doing so at the end of the follow up period, but additional fluids and semi-solids were given before the recommended time. Only two (10.5%) mothers breastfed exclusively from birth to 6 months. Prelacteal feeds, home remedies and traditional medicine bought from traditional healers were given by over a third of the mothers in the first week of life. Concern over babies’ bowel habits and interpretation of persistent crying as abdominal colic underlay several mothers receiving and following advice to give non-prescribed treatments such as gripe water and traditional medicines. Early introduction of maize porridge from 3 months of age as a complementary food, because of perceived hunger of the child, was recommended by other family members.

A key finding in our study was that additional fluids were commonly given for treatment purposes in the neonatal period, particularly in the first week. This appeared to be driven by family members’ perception of gut problems prompting treatment with traditional herbal infusions, orange juice or sugar and salt solution for the babies to open their bowels. Another study of caregivers’ concerns on infant health and nutrition problems carried out about 100 km south of our study area reported that abdominal symptoms were the most commonly mentioned problem, however the timing of treatment was not elicited in their study [25]. Additional fluids in the early neonatal period may alter the constituents of the gut microbiome, affect priming of the gut-associated immune system and increase risks of diarrhoeal disease [26].

Apart from two reported instances where nurses advised mothers to use gripe water we did not find health workers’ advice to counter the message for exclusive breastfeeding, however the existence of a prolonged nurses’ strike during the data collection period reduced opportunities for consultation. This differs from a study in South Africa where nurses were reported to give prelacteal feeds in facilities and offer inappropriate feeding advice [27].

As found in other African studies, porridge was introduced to infants before the recommended time of 6 months, mainly at grandmothers’ and neighbours’ suggestions [28, 29]. The reasons for introduction were similar to those found in other qualitative studies: to calm the baby, to reduce hunger and to teach the baby about different foods [28, 30]. Participants in our study were young and those who were married were under pressure to obey their mother-in-law’s advice else they faced the possibility of being returned to their parental home [12].

Crying in the first few days of life led to concerns that the volume of colostrum was insufficient and the baby was at risk of starving, hence prelacteal feeds were given when the emphasis should have been on frequent breastfeeding and learning correct positioning and attachment to increase milk supply. As the baby grew older, crying after feeds and appearing to be hungry were stated as the main reasons for giving additional fluids and food. Failure to latch onto the breast adequately can result in slow milk flow, with rapid sucking and increased effort from the baby, yet mothers were unaware of this potential cause and attributed their perceived insufficient milk to poor diet, as has been found in other Kenyan studies [31, 32].

Although the sample size was small, the breastfeeding observation aid clearly showed that poor position and attachment were ongoing issues for mothers. Few mothers reported any advice on breastfeeding technique from health workers either immediately after the delivery or at routine clinic visits. Researchers counselled the mothers with breastfeeding difficulties and referred them to health facilities when this impaired infant feeding but the persistence of problems in positioning implies that there may be conflicting advice from other sources in the community. Fears of suffocating the baby with the breast blocking the baby’s nose if held too close during breastfeeding had been noted in a recent study in the same location [12].

### Strengths of the study

Strengths of our study are that repeated home visits enabled the researchers to develop trust and open communication and to detect transition timepoints between exclusive and non-exclusive breastfeeding.

Interviews were led by the Kenyan researcher who came from a similar background to the participants. Repeated interviews allowed the participants to build a rapport with both researchers.

Direct observation of breastfeeds was acceptable to all participants and enabled analysis of problems with technique which are potentially modifiable.

We considered the potential for social desirability bias and emphasized to the participants that we respected their honest answers and that their data was confidential. We felt that conducting the interviews away from other family members would enable participants to answer more freely, but we gave them the option to allow family members to stay. The setting in their home rather than clinic was chosen to reduce the likelihood of giving “medically correct” answers. We were able to triangulate answers with our observations of infant feeding in the home setting.

### Limitations of the study

Our study had a small sample size and restricted itself to first-time mothers living in a single geographical location therefore the results may not be generalizable to other populations, however the lack of autonomy of teenage mothers over infant feeding decisions has been described in other African settings, highlighting the need for a more family-centred approach in promotion of exclusive breastfeeding [33]. Another potential limitation is that recurrent research visits may have changed infant feeding behaviour. There was a risk of social desirability bias in that the mothers knew that the study was researching barriers to exclusive breastfeeding and so may have reported that they were practicing EBF when they were not.

The study took place during several prolonged government nurses’ and doctors’ strikes which meant that the government dispensary and county hospital which serve the local population were closed for 5 months and several babies did not receive immunizations. This may also have led to greater use of traditional and home remedies as some families did not have sufficient funds to seek treatment from trained health workers in private health facilities. The proportion of women who gave birth at home may also have been greater than usual with the potential for increased risk of infection in mother and baby.

### Recommendations

Despite this caution it appears that the first week of life is a period when many challenges to exclusive breastfeeding occur. In this context there is a need to devise strategies for contacts with pregnant women and their home-based advisers starting in the antenatal period to preempt the use of non-prescribed medicines and support mothers to follow recommended infant feeding practices. Prior to delivery, awareness should be raised of the role of immediate skin-to-skin contact after birth in establishing early breastfeeding and teaching on the importance of good attachment to the breast to stimulate milk production. As well as dialogue, use of visual aids such as flipcharts, dolls and videos is recommended to demonstrate correct positioning and attachment. Pictures and videos, that have been checked so as to be relevant to the local context and interpretable by low-literacy populations, can be a useful adjunct to health education messages and can improve attention to, and recall of information [34, 35].

In the early postnatal period, counselling of mothers and problem-oriented support for breastfeeding ideally should take place in their homes but lack of geographic coverage by active community health volunteers in the study area limits the possibility of home visits. Health professionals should use the opportunities at routine clinic visits for BCG immunization (commonly given within the first few weeks of life) and mothers’ postnatal checkups to proactively ask about breastfeeding concerns with the mother and their accompanying relatives, and provide support when problems are found. Health workers and local health committees may also play a role in lobbying schools and workplaces to provide facilities for mothers to breastfeed or express milk during breaks.

First-time mothers in our study received advice from other family members and neighbours to supplement breastmilk with other fluids and foods when babies cried, so future research should seek to understand in more depth, caregivers’ perception of and responses to infant negative emotional states and hunger cues.

## Conclusions

Exclusive breastfeeding from birth to 6 months was uncommon. Modifiable barriers to exclusive breastfeeding occur particularly in the early postnatal period. Health workers should aim to enable first-time mothers with skills and knowledge antenatally and support optimal feeding as perinatal challenges arise. Ensuring capacity for detection and support of mothers with breastfeeding problems at child welfare clinic attendances and other contacts with health service providers should be prioritized in order to help improve health and growth from early in life. Older female relatives are key stakeholders in household infant feeding and treatment seeking decisions and must be included in interventions to change breastfeeding norms.

## Supplementary information

**Additional file 1.** Breastfeeding observation aid. Checklist for assessment of breastfeeding technique.

**Additional file 2.** Interview topic guide. List of topics for interviews of mothers at home visits 1 to 9.

## Data Availability

The dataset is available from the corresponding author on reasonable request.

## Abbreviations

BCG: Bacillus Calmette Guerin
BOA: Breastfeeding observation aid
CGMR-C: Centre for Geographic Medicine Research Coast
EBF: Exclusive breastfeeding
KEMRI: Kenya Medical Research Institute
SERU: Scientific and ethics review unit
UNICEF: United Nations Children’s Fund
WHO: World Health Organization
