# Supplementary material for: Exclusive breastfeeding in first-time mothers in rural Kenya: a longitudinal observational study of feeding patterns in the first six months of life
Source: Int Breastfeed J. 2020 Mar 5;15:17. doi: 10.1186/s13006-020-00260-5 (PMC7059377; doi:10.1186/s13006-020-00260-5)
Supplement: Supplementary file 1 — Additional file 1. Breastfeeding observation aid. Checklist for assessment of breastfeeding technique. [file 13006_2020_260_MOESM1_ESM.pdf]

BREASTFEED OBSERVATION AID

Mother's name ______________________________ Date __________________

Baby's name ________________________________ Baby's age ______________

Signs that breastfeeding is going well:	Signs of possible difficulty:
**GENERAL**	
Mother:	*Mother:*
☐ Mother looks healthy	☐ Mother looks ill or depressed
☐ Mother relaxed and comfortable	☐ Mother looks tense and uncomfortable
☐ Signs of bonding between mother and baby	☐ No mother/baby eye contact
Baby:	*Baby:*
☐ Baby looks healthy	☐ Baby looks sleepy or ill
☐ Baby calm and relaxed	☐ Baby is restless or crying
☐ Baby reaches or roots for breast if hungry	☐ Baby does not reach or root
**BREASTS**	
☐ Breasts look healthy	☐ Breasts look red, swollen, or sore
☐ No pain or discomfort	☐ Breast or nipple painful
☐ Breast well supported with fingers away from nipple	☐ Breasts held with fingers on areola
☐ Nipple protractile	☐ Nipple flat, not protractile
**BABY'S POSITION**	
☐ Baby's head and body in line	☐ Baby's neck and head twisted to feed
☐ Baby held close to mother's body	☐ Baby not held close
☐ Baby's whole body supported	☐ Baby supported by head and neck only
☐ Baby approaches breast, nose to nipple	☐ Baby approaches breast, lower lip/chin to nipple
**BABY'S ATTACHMENT**	
☐ More areola seen above baby's top lip	☐ More areola seen below bottom lip
☐ Baby's mouth open wide	☐ Baby's mouth not open wide
☐ Lower lip turned outwards	☐ Lips pointing forward or turned in
☐ Baby's chin touches breast	☐ Baby's chin not touching breast
**SUCKLING**	
☐ Slow, deep sucks with pauses	☐ Rapid shallow sucks
☐ Cheeks round when suckling	☐ Cheeks pulled in when suckling
☐ Baby releases breast when finished	☐ Mother takes baby off the breast
☐ Mother notices signs of oxytocin reflex	☐ No signs of oxytocin reflex noticed

Notes:
