# Supplementary material for: Exclusive breastfeeding in first-time mothers in rural Kenya: a longitudinal observational study of feeding patterns in the first six months of life
Source: Int Breastfeed J. 2020 Mar 5;15:17. doi: 10.1186/s13006-020-00260-5 (PMC7059377; doi:10.1186/s13006-020-00260-5)
Supplement: Supplementary file 2 — Additional file 2. Interview topic guide. List of topics for interviews of mothers at home visits 1 to 9. [file 13006_2020_260_MOESM2_ESM.docx]

**Interview topic guide**

**Visit 1 - late pregnancy**

**Birth plans and help with baby**

Where do you plan to give birth?

Who will help to look after you and the baby in the early days after the birth? How have you chosen this person?

How long do you think they will help you?

What help do you think you will need?

**Breastfeeding intentions and knowledge**

How do you plan to feed the baby?

Have you had anyone explain how to breastfeed?

Do you know who to ask for advice in case of any breastfeeding problems?

**Complementary feeding**

When do you plan to introduce other foods to the baby? What foods will you give to start with? When will you give the baby normal family food?

**Work and childcare intentions**

If planning to work outside the home or return to school, when will you go back? Who will look after the baby whilst you are at work/school? What will the baby feed on while you are at work/school?

**Expressed breastmilk**

Have you been taught how to express breastmilk?

Do you intend to give your baby expressed breastmilk?

**Mental and physical problems**

Have you had any health concerns during this pregnancy? If yes, probe.

How is your mood? *(administer PHQ-9)*

**Family planning intentions**

Have you thought about which family planning method to use after the baby is born?

When do you intend to start using family planning?

Which method do you prefer to use?

**Visit 2 at 1 week post-birth**

**Birth details and mother’s health**

Collect information on delivery and postnatal checkup

Delivery: place, date, time, type (SVD/assisted vaginal/LSCS) Any complications?

If home birth, name of midwife, relationship to mother.

Postnatal check-up at clinic? Date of postnatal visit.

Community health worker visit? If yes, by whom, date?

Any health concerns?

**Household and visitors**

Family circumstances changed?

Help around time of delivery

Who is helping you to look after the baby at home?

Is s/he a household member or visiting? If visiting, how long will she stay?

How does she help you with the baby?

Have you travelled away from home since the birth? (*probe- reasons, length of stay, relatives etc.)*

**Baby’s data and health**

Baby details: Gender. Birth weight or if not known then 1^st^ recorded weight and when. Gestation. Singleton/multiple birth- birth order. Congenital anomalies

First time baby held and first feed: how long after birth? Prelacteal feeds? Reasons for giving?

Has your baby been well?

If not, what was the problem and how did you manage it? (*probe for treatment seeking behaviour, treatment given, by whom, problem resolved satisfactorily?*)

Any other concerns about the baby?

**Feeding, adequacy and problems**

Is your baby calm when s/he feeds?

How many times have you breastfed in the last 24 hours?

How many dirty/wet nappies in the last 24 hours?

Is your baby gaining weight OK?

How are you finding breastfeeding? Any problems? (*probe for nipple problems, amount of milk, abscesses; advice/treatment sought/offered, by whom)*

Have you or anyone else given the baby any fluids apart from your milk? *If yes, probe type of fluid, number of times given, reasons why, who gave the fluids.*

Have you or anyone else given the baby any food apart from your milk? *If yes probe type of food, number of times given, reasons why, who gave the food.*

**Visits 3-9**

How are you and your baby? How are your husband and family? Any changes in your household since my last visit? (*probe for changes in marital status if appropriate)*

Who is helping you to look after the baby at home?

Is s/he a household member or visiting? If visiting, how long will she stay?

How does she help you with the baby?

Any concerns (*probe*)

Have you been to clinic since last visit? (*Probe where and reason (routine or for a problem), advice/ treatment given, immunizations, postnatal checkup etc.)*

**Breastfeeding adequacy**

Is your baby calm when s/he feeds?

Are you comfortable when you are breastfeeding (if no- *probe for problems*)

How many times have you breastfed in the last 24 hours?

How many dirty/wet nappies in the last 24 hours?

Is your baby gaining weight OK?

**Co-sleeping**

Where did your baby sleep last night? With whom? What led to this sleeping arrangement?

**Breastfeeding problems**

How are you finding breastfeeding? Any problems? (*probe for nipple problems, amount of milk, abscesses; advice/treatment sought/offered, by whom)*

Have you or anyone else given the baby any fluids apart from your milk? *If yes, probe type of fluid, number of times given, reasons why, who gave the fluids.*

Have you or anyone else given the baby any food apart from your milk? *If yes probe type of food, number of times given, reasons why, who gave the food.*

**Baby’s health**

Is your baby growing well? How is your baby’s growth measured?

Has your baby been well since my last visit?

If not, what was the problem and how did you manage it? (*probe for treatment seeking behaviour, treatment given, by whom, problem resolved satisfactorily?*)

Any other concerns about the baby?

**Mother’s health and family planning**

Have you had any health concerns since my last visit? *If yes*, *probe for treatment seeking behaviour, treatment given, by whom, problem resolved satisfactorily?*

Is anything causing you mental stress? How is your mood? *(administer PHQ-9)*

Have your periods returned yet? Have you resumed sexual activity with your partner? Are you using contraception? *If yes, probe method of contraception used*
